# Association between caregiver quality of life and the care provided to persons with Alzheimer’s disease: protocol for a systematic review

**DOI:** 10.1186/2046-4053-2-17

**Published:** 2013-03-13

**Authors:** Afeez Abiola Hazzan, Jenny Ploeg, Harry Shannon, Parminder Raina, Mark Oremus

**Affiliations:** 1 Department of Clinical Epidemiology and Biostatistics, McMaster University, Hamilton, ON, Canada; 2 School of Nursing, Faculty of Health Sciences, McMaster University, Hamilton, ON, Canada

**Keywords:** Alzheimer’s disease (AD), Caregiver, Quality of life (QoL), Level of care, Quality of care, PROSPERO registration number: CRD42013003613

## Abstract

**Background:**

Primary informal caregivers provide a substantial amount of the care and support for persons with Alzheimer’s disease (AD). This review aims to investigate the association between the quality of life (QoL) of primary informal AD caregivers and the level of care that these caregivers provide to persons with AD.

**Methods:**

Studies involving primary informal caregivers of persons with AD will be included in the review. These studies will be required to focus on the care that caregivers provide for their loved ones. The primary outcome is level or quality of care. The main independent variable is caregiver QoL. In addition to QoL, we will include studies that examine other independent variables that are considered to be important components of QoL. These variables include social support, caregiver burden, caregiver wellbeing, and caregiver depression.

We will search Medline-OVID, Embase-OVID, Cochrane Central-OVID, and PsycINFO-OVID from inception onwards. Two raters will independently screen each article using pre-established inclusion/exclusion criteria. Screening will take place at two levels: title and abstract, and full text. Conflicts will be resolved by discussion or by a third reviewer. We will assess the risk of bias of each included study using standardized quality assessment tools for specific types of designs. A narrative synthesis method will be used to describe our findings. Quantitative summary and meta-analysis will be conducted if appropriate. We will employ GRADE to evaluate the strength of the evidence in this review.

**Discussion:**

Results of this systematic review will show whether and how caregiver QoL is related to the level of care that caregivers provide to persons with AD.

## Background

Alzheimer’s disease (AD) is a neurodegenerative disorder characterized by progressive declines in cognitive and functional abilities. Symptoms often begin with memory loss and progress to an inability to perform basic activities of daily living (for example, bathing and eating) [1]. The estimated prevalence of AD in the Canadian population aged 65 years or over is 300,000 persons [2]. Approximately 40,000 Canadians develop the disease annually [3]. Projections suggest the total number of Canadians with AD could rise to 509,000 in 2031 [3,4]. In the United States, an estimated 5.4 million people have AD in 2012, including 5.2 million people age 65 and older [5]. About 35 million people worldwide had AD in 2010 and this figure has been projected to top 115 million by 2050 [6]. There will be a 63% increase in the proportion of people living with dementia in North America over the next 20 years [6].

Primary informal caregivers provide a substantial amount of the care and support required for persons with AD. These caregivers are usually close relatives (for example, spouses, children) of their ‘loved ones’ with AD and they are generally unpaid for the care they provide. As AD progresses, caregivers watch their loved ones deteriorate at the same time as they are called upon to perform an increasing range of tasks that ultimately include helping loved ones with basic activities such as toileting and dressing [7]. The quality of life (QoL) experienced by unpaid family caregivers in AD has been shown to be lower than the QoL of caregivers for persons who do not have AD [7]. Among carers of people with chronic conditions in general, caring for a person with AD is particularly stressful, and these caregivers have been called the ‘hidden victims’ of the disease [6,8]. The strain of performing caregiver tasks, coupled with emotional stress and a sense of being ‘trapped’ in the caregiving role, are among the leading reasons caregivers cite for institutionalizing their loved ones [9].

Research has shown that lower QoL can increase absences from work and reduce job productivity [10]. In the domain of care provision, it is possible that these workplace productivity issues might translate into declining ‘caregiver productivity’. Therefore, this review aims to investigate the association between the QoL of primary informal AD caregivers and the level of care that these caregivers provide to persons with AD. This review will address two research questions:

1. What is the relationship between caregiver QoL and level of care?

2. What is the relationship between caregiver QoL and quality of care?

Level of care includes caregiver willingness to provide care and the amount of time spent providing care. Quality of care will be operationalized using any means (caregiver mastery, task management strategy, and so on) that have been designed to measure this concept [11].

In addition to QoL, we will include studies examining related concepts such as social support, caregiver burden, wellbeing, and caregiver depression. These concepts are closely related to QoL and could provide valuable insight into the relationship between QoL and level of care.

## Methods

The systematic review described in this protocol follows the PRISMA statement [12].

### Information sources and literature search

The following electronic databases will be searched from start date to 2012: Medline-OVID, Embase-OVID, Cochrane Central-OVID, and PsycINFO-OVID. Internet searches for gray literature will also be performed.

#### General search terms for electronic databases

The following terms will be used when devising search strategies for electronic databases:

1. *Alzheimer Disease/

2. Family Nursing/

3. 1 and 2

4. (family adj2 caregiv*).ti.

5. 1 and 4

6. *Caregivers/

7. 1 and 6

8. ((informal or family or spous*) adj3 (care* or caregiv*)).tw.

9. 7 and 8

10. 3 or 5 or 9

11. Alzheime*.ti.

12. ((informal or family or spous*) and (care* or caregiv*)).ti.

13. 11 and 12

14. 9 or 13

15. limit 14 to (biography or comment or editorial or in vitro or letter)

16. 14 not 15

The search strings above will be adjusted for each database. An experienced librarian will conduct the literature search in all four databases. Results from the literature search will be uploaded to DistillerSR (Evidence Partners Incorporated, Ottawa, Canada; Copyright 2010), an online application that will be used for the screening and data extraction phases of this systematic review.

### Eligibility criteria

We will include studies dealing with primary informal caregivers of community-dwelling persons with AD. These unpaid caregivers are typically close relatives (for example, spouses, children) who provide the bulk of care and support. Studies including only paid caregivers, such as home help aides or employees of long-term care facilities, as well as studies set only in institutions, will be excluded from the review. There will be no restrictions on the sex, age, or ethnic background of caregivers.

Included studies must report at least one of the following outcomes in relation to caregiver QoL: level of care, quality of care, amount of time spent providing care, and similar variables dealing with caregiver performance in their caregiving role. We will include primary studies that provide quantitative results. We will include studies written in any language that are experimental (including RCTs, quasi-randomized trials, controlled clinical trials), quasi-experimental (including interrupted time series and controlled before and after studies), or observational designs with comparison groups.

#### Level or quality of care

Due to the difficulty involved in determining what constitutes ‘quality’ care, studies using any scale that was designed to measure quality will be considered for inclusion. This includes studies measuring ‘level’ or ‘quality’ of care from a task-oriented perspective.

#### Quality-of-life (QoL)

The concept of QoL is multi-faceted and may be defined in several ways. In this review, QoL will be treated as an independent variable. The most common approach to measuring QoL involves the use of scales, several of which have been developed for healthcare research [13]. Popular, generic, health-related QoL scales include the Short Form 36 (SF-36) [14], EuroQoL Group’s EQ-5D [15], and the World Health Organization Quality of Life-BREF (WHOQOL-BREF) [16]. Other QoL scales, such as the Quality-of-life - Alzheimer’s Disease (QOL-AD) scale [17], were specifically developed for use in patient populations or diseases.

We will include any study measuring QoL, regardless of the specific scale used. In addition to QoL, studies examining the impact of the following constructs on our outcomes will be included.

#### Social support

Social support is a multidimensional construct of the extent to which individuals receive emotional support, instrumental assistance, information, guidance and feedback, personal appraisal support, and companionship from family members, friends, co-workers, other persons (for example, acquaintances, religious leaders, therapists), or organizations (for example, caregiver support groups).

#### Caregiver burden

Caregiver burden is operationalized by any construct representing the physical, emotional, and financial cost of providing care for a loved one with AD. The Zarit Burden Interview (ZBI) [8,18] is a widely used instrument for measuring caregiver burden.

#### Caregiver depression

Although depression is an element of QoL, it is also an important construct on its own. Indeed, depression is one of the common side effects of long-term caregiving [19]. Depression can be measured by several instruments including the Center for Epidemiologic Studies-Depression (CES-D) scale [20,21].

We will include any study measuring these constructs, regardless of the means by which these constructs are measured.

Included studies may present quantitative results only.

#### Selection procedure

Two reviewers will independently screen the titles and abstracts of studies identified in the literature search. Studies meeting the eligibility criteria, or studies whose titles and abstracts do not provide sufficient information to assess eligibility, will advance to full-text screening. During full-text screening, the reviewers will independently read entire papers and assess eligibility. Conflicts will be resolved by discussion between the two reviewers or by the involvement of a third reviewer.

#### Data collection process

A detailed data collection form will be developed to collect information about study characteristics (study design, sample size, year, country), participant characteristics (for example, type and number of patients, caregiver relationship to person with AD, living situation, age, mean and standard deviation, AD diagnosis criteria), and results (for example, quality of life, quality of care, institutionalization, amount of care, and so on).

Data will be extracted using an online form uploaded to DistillerSR. The data extraction form will be piloted by two reviewers and further refined if necessary. Two reviewers will independently extract all of the data to achieve the highest level of accuracy. Reviewers will meet to resolve discrepancies. If the two reviewers cannot agree, then a third reviewer will be asked to adjudicate.

In cases where studies report outcome results over different time periods, we will extract data from each time period to examine the impact of the intervention over time. Further, in cases where multiple publications report data from the same study, we will use the most current report of the outcomes of interest. When data are not clearly reported, we will contact the lead author of the study for clarification.

#### Assessment of risk of bias

Each included study will be assessed for risk of bias using appropriate quality assessment tools. For studies employing RCT design, we will use the Cochrane Risk of Bias Tool [22]. The Cochrane Effective Practice and Organisation of Care Risk of Bias Tool will be used for assessment of risk of bias for controlled clinical trials, interrupted time series, and controlled before-after studies [23,24]. Finally, the Newcastle-Ottawa Scale will be used for studies employing cohort and case control designs [25].

We will use GRADE to assess the level of evidence across studies and also use funnel plots to assess publication bias [26].

#### Synthesis

A narrative synthesis method will be used to describe the results. All included studies will be summarized in narrative form, and summary tables will be created showing key study characteristics (that is, population characteristics, treatment interventions, study outcomes, sample sizes, settings, funding sources, and comparator treatments (type, duration, and provider)), methodological limitations, and any other important aspect related to each research question of interest.

Meta-analysis will be performed if possible from the data extracted from the included studies. If clinical groups are too heterogeneous to permit meta-analysis, a separate qualitative analysis will be presented and graphical representation may be used to display main study outcomes.

If outcomes of interest in each included study were reported using different outcome measures on a continuous scale, the DerSimonian and Laird random effects models with inverse variance method will be utilized to generate the summary measures of effect in the form of standardized mean difference (SMD) for each outcome [27]. The use of SMD as a summary statistic in a systematic review is appropriate if the same oucome (that is, quality of care) is assessed in a variety of ways or if different psychometric scales were used [27]. In such a situation, it would be necessary to standardize the results of the studies before they could be compared across studies or combined in a quantitative synthesis.

Standardized mean differences (SMDs) will be calculated using change from baseline data, that is, mean difference between pre-treatment (baseline) and post-treatment (final/endpoint) scores along with its standard deviation for both intervention and control groups. Appropriate correlation between pre-treatment (baseline) and post-treatment (final/endpoint) level of care scores will be used based on evidence from existing literature. The Cochran’s Q (α=0.10) and I^2^ statistic will be utilized to quantify the statistical heterogeneity between studies examining our outcomes of interest, where *P* <0.10 indicates a high level of statistical heterogenity between these studies. Sensitivity analyses will be performed on the type of intervention, study risk of bias and by removing studies with obvious between-group baseline imbalance in order to evaluate statistical stability and effect on statistical heterogeneity.

## Discussion

Results of this systematic review will show whether and how caregiver QoL is related to the level of care that caregivers provide to persons with AD. The results could help caregivers, caregiver support organizations, and policy-makers make informed decisions about programs to optimize the care that persons with AD receive from their caregivers.

We will publish results of this systematic review in a peer-reviewed journal to make the results widely available to researchers and policy-makers. We will also present our results at relevant research meetings and make the findings accessible to caregiver support organizations.

## Competing interests

The authors declare that they have no competing interests.

## Authors’ contributions

AAH conceived the study, designed the study, and helped write the draft protocol. MO provided input to the conception and design of the study, and helped draft the protocol. JP, HS, and PR provided input to the design of the study and draft of the protocol. All authors read and approved the final protocol.
